# Physical collection and viability of airborne bacteria collected under electrostatic field with different sampling media and protocols towards rapid detection

**DOI:** 10.1038/s41598-021-94033-7

**Published:** 2021-07-16

**Authors:** Seongkyeol Hong, Myeong-Woo Kim, Jaesung Jang

**Affiliations:** 1 Department of Mechanical Engineering, Ulsan National Institute of Science and Technology (UNIST), Ulsan, 44919 Republic of Korea; 2 Department of Biomedical Engineering, UNIST, Ulsan, 44919 Republic of Korea; 3 Department of Urban and Environmental Engineering, UNIST, Ulsan, 44919 Republic of Korea

**Keywords:** Environmental sciences, Health care, Engineering

## Abstract

Electrostatic samplers have been increasingly studied for sampling of viral and bacterial aerosols, and bioaerosol samplers are required to provide concentrated liquid samples with high physical collection and biological recovery, which would be critical for rapid detection. Here, the effects of sampling media and protocols on the physical collection and biological recovery of two airborne bacteria (*Pseudomonas fluorescens* and *Micrococcus luteus*) under electrostatic field were investigated using a personal electrostatic particle concentrator (EPC). Deionized (DI) water with/without sodium dodecyl sulfate (SDS) and phosphate buffered saline were tested as sampling media. A polystyrene container was mounted onto the collection electrode of the EPC for stable storage and vortexing after capture. Many bacterial cells were found to be deposited on the bottom surface of the container submerged in the media via electrophoresis, and among the tested sampling protocols, wet sampling with a container and subsequent vortexing offered the most bacteria in the collection suspension. Experiments with several sampling media showed that 0.001–0.01% SDS-DI water demonstrated the highest recovery rate in the EPC. These findings would be valuable in the field of sampling and subsequent rapid detection of bioaerosols.

## Introduction

Bioaerosols such as airborne viruses and bacteria can be transmitted and spread through the air rapidly and widely. These biological particles, especially pathogenic ones, can cause infectious diseases and other adverse health effects^1^. The identification of airborne biological particles is usually made through air sampling and subsequent analysis, such as cultivation, polymerase chain reaction, immunoassay, etc.^2^, and most of the bioanalysis techniques are performed in the liquid phase. Considering the detection limits of the analysis techniques and equipment, an air sampler is highly required to provide concentrated liquid samples from low concentrations of biological particles in the air. Furthermore, the physical and/or biological states of the particles need to be preserved during sampling, depending on subsequent detection or analytical methods^3^.

Physical collection and viability of airborne bacteria depend strongly on their sampling methods. Impactors, impingers, and cyclone samplers have been commonly used for sampling of bacterial aerosols. Because these samplers are based on the inertial impact of airborne particles, high collection efficiencies can be obtained if large sampling velocities are used. However, the mechanical stress caused by the large sampling velocities may result in significant damage to the sampled bacteria^4^ and even to their DNAs^5^. Recently, electrostatic samplers have been increasingly studied for sampling of bacterial aerosols^6–13^, utilizing electrostatic attraction of pre-charged bacterial particles onto wet or dry electrodes. Collection efficiencies of the electrostatic samplers were high for a wide range of particle sizes, although they decreased as the sampling velocity increased. In addition, airborne biological particles can be highly concentrated into a small amount of liquid using a concentrating electric field configuration^14^, which may be beneficial for direct and rapid on-site detection of bioaerosols, and low sampling velocities in the electrostatic samplers can provide a basis for high biological recovery of bacteria. Zhen et al*.*^15^ showed that the membrane damage of bacterial cells was the lowest in their electrostatic sampler compared with the BioSampler (impinger), BioStage (impactor), and Button Sampler (filter), because of the lowest sampling velocity in the electrostatic sampler.

Since bacterial viability is vulnerable to dehydration^3,16,17^, wet-phase sampling is usually preferred. Furthermore, it is critical to determine how sampling media on the collection electrode affect sampled bacteria and what sampling media may be the most appropriate for sampling of bacteria. Impactors usually utilize moisture-containing agar plates to collect and culture airborne bacteria, and sampling periods in the impactors should be short enough to prevent dehydration of the agar surfaces via air flow^18^. Deionized (DI) water and phosphate-buffered saline (PBS) are commonly used in impingers as sampling media^19^, and peptone water with antifoaming agents and surfactant such as Tween 80 is used for enhanced cell collection^19,20^. Mineral oil could be used for long-term stable sampling in impingers to minimize evaporation although extra extraction is required^21^. These sampling media used in impingers or wet cyclones can also be used as extraction liquids in dry-phase samplers. PBS supplemented with antifoam A and surfactant of Triton X-100 was used as an extraction liquid in an electrostatic sampler, and electret filters^3^. Sampling media are presumed to play an important role in the wet-phase electrostatic sampling of airborne bacteria, since the liquid can also act as an electrolyte and may electrically affect the suspended, charged bacteria in the presence of electric field.

In this study, the effects of various sampling media and protocols on physical collection and biological recovery of airborne bacteria under electrostatic field were investigated using the personal electrostatic particle concentrator (EPC) and were compared with the standard impinger, BioSampler. The EPC was previously developed for the purpose of on-site detection of airborne viruses in conjunction with microfluidics-based^22^ and paper-based sensors^23–25^. The recovery rate and the concentrations of MS2 and T3 bacteriophages collected in the EPC were remarkably higher than those in the BioSampler, which was attributed to sampling velocity three orders of magnitude lower in the EPC^26^. Here, several sampling characteristics of gram negative (*Pseudomonas fluorescens*) and gram positive (*Micrococcus luteus*) bacterial aerosols were evaluated using the EPC and the BioSampler.

## Results and discussion

### Electrophoretic deposition of bacterial cells

Effective capture and detachment of airborne particles is critical for electrostatic sampling and subsequent detection. Most electrostatic samplers involve large-area collection electrodes to increase collection efficiencies; hence, the collection spots in the samplers are not small enough to be conveniently and well vortexed for detachment, and collected liquid samples are forced to flow to storage places. In contrast, a small collection container was used in this study, allowing vortexing. Here, four sampling protocols for electrostatic sampling and detachment using the EPC were evaluated. They include Wet-w-cont-NV (Wet sampling with a container (cont), however, no vortexing after the sampling), Wet-w-cont-V (Wet sampling with a container and subsequent vortexing), Wet-w/o-cont-NV (Wet sampling on a polyimide film attached onto the collection electrode of the EPC instead of a container, and no vortexing), and Dry-w-cont-V (Dry sampling with a container and subsequent vortexing after buffer injection into the container).

**Figure 1:**
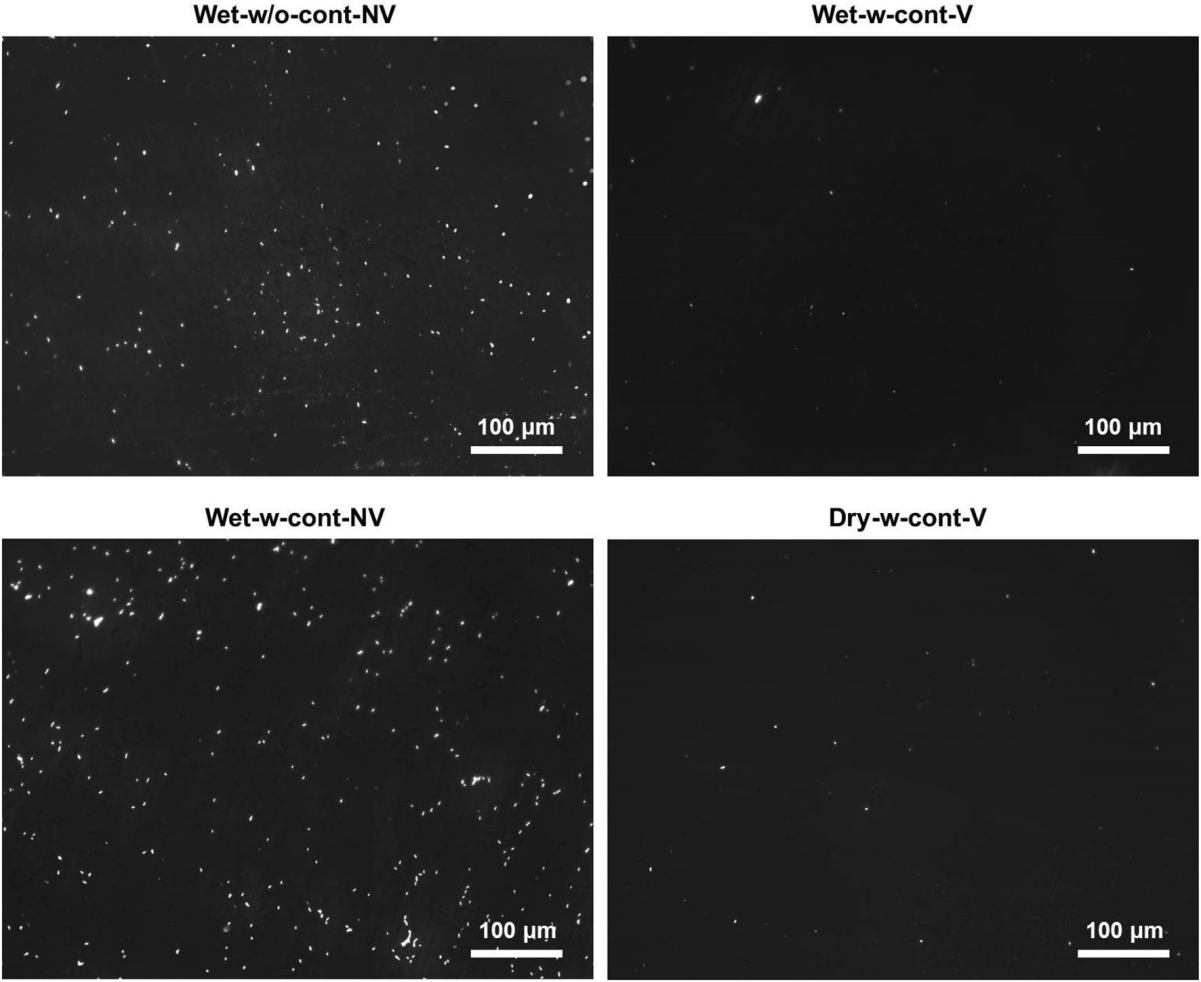
Representative fluorescence micrographs of *P. fluorescens* deposited on the surfaces of plastic containers and a thin polyimide film for a case of Wet-w/o-cont-NV after removing media. Four different sampling protocols were used in the EPC (− 5 kV) with a sampling medium of 1 × phosphate buffered saline: Wet-w-cont-NV (Wet sampling with a container, but no vortexing after the sampling), Wet-w-cont-V (Wet sampling with a container and subsequent vortexing after the sampling), Wet-w/o-cont-NV (Wet sampling on a polyimide film of the EPC electrode with no vortexing afterwards), and Dry-w-cont-V (Dry sampling with a container and subsequent vortexing after buffer addition). A great number of bacterial cells were attached to the surfaces when no vortexing was applied after electrostatic sampling (Wet-w/o-cont-NV and Wet-w-cont-NV).

It was observed in this study that many bacteria were attached to the bottom surfaces of the container (Wet-w-cont-NV) and the polyimide film (Wet-w/o-cont-NV) when no vortexing was applied after electrostatic collection (Fig. 1). This was attributed to electrophoresis of charged bacteria in aqueous solutions under the presence of electric field. When a charged particle is suspended in an electrolyte, the Coulomb force is exerted on the particle with the surface charges, whereas the electrophoretic retardation force was applied to the ions in the diffuse layer and the drag force is exerted in the opposite direction to the particle’s movement caused by the Coulomb force. That is, the particle migrates according to the direction of the electric field and the charges on the surface. Mainelis et al*.*^17^ also observed similar phenomenon; however, their results were based on colony enumeration alone, and the physical deposition of bacteria was not quantified, underestimating the total number of bacteria collected under electrostatic sampling.

### Influences of sampling protocols

**Figure 2:**
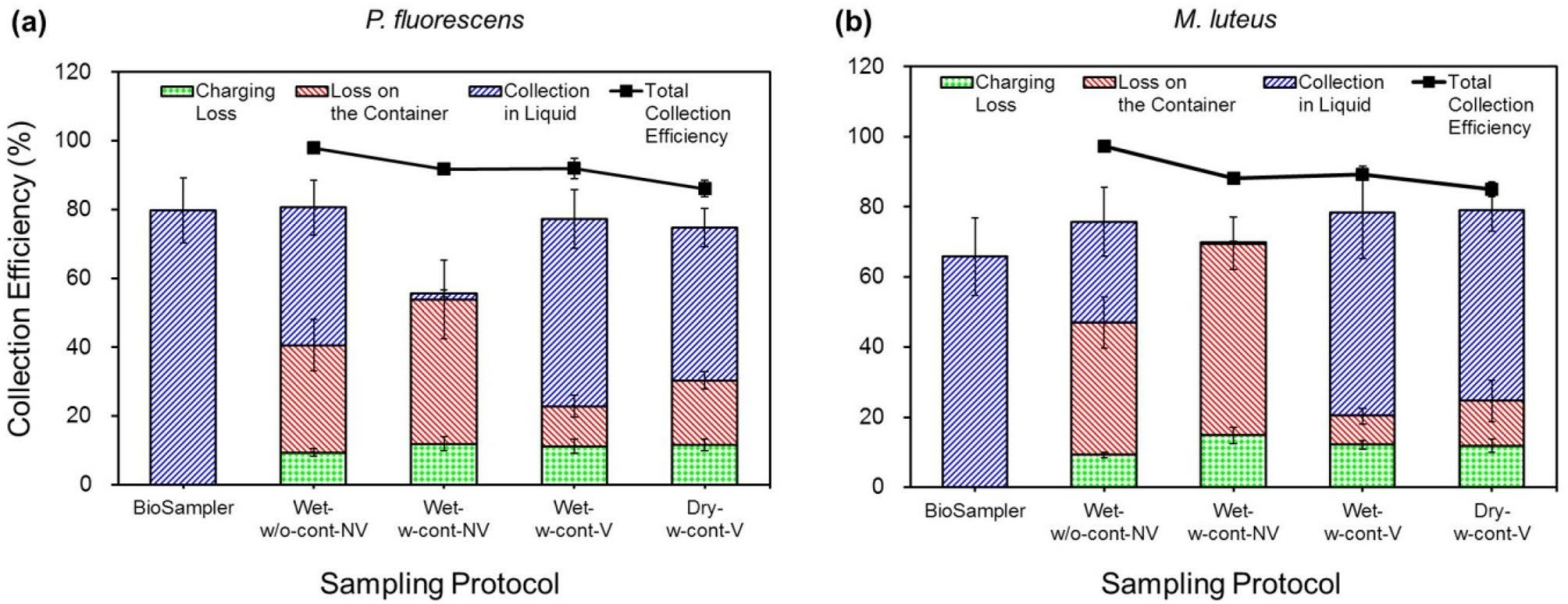
Collection efficiencies of (**a**) *P. fluorescens* and (**b**) *M. luteus* in the EPC (− 5 kV) using different sampling protocols and in the BioSampler with a sampling medium of 1 × phosphate buffered saline. The total collection efficiencies were compared with the fractions of bacterial cells remaining in the EPC when four different sampling protocols were used. The Loss on the Container in case of Wet-w/o-cont-NV indicates the particle losses attached to a thin polyimide film. Wet-w-cont-NV (Wet sampling with a container, but no vortexing after the sampling), Wet-w-cont-V (Wet sampling with a container and subsequent vortexing after the sampling), Wet-w/o-cont-NV (Wet sampling on a polyimide film of the EPC electrode with no vortexing afterwards), and Dry-w-cont-V (Dry sampling with a container and subsequent vortexing after buffer addition).

The physical collection efficiencies based on fluorescence micrographs and optical particle counter (OPC) measurements are shown in Fig. 2, and similar trends were observed between *P. fluorescens* and *M. luteus*. The same plastic containers were used for the three sampling protocols using a container, and the total collection efficiencies for the three sampling protocols were lower than that for Wet-w/o-cont-NV, which was due to the reduced electric field intensity owing to the plastic containers. Moreover, the intrinsic collection efficiencies, which consider particle collection within sampling media alone, in the EPC were smaller than those in the BioSampler, because of particle losses in the corona charger, which is referred to as charging loss, and the electrophoretic deposition of particles on the container, which is referred to as loss on the container. The charging losses were observed to be 9.1–14.9%.

It was also observed that the intrinsic collection efficiencies in case of Wet-w/o-cont-NV were higher than those in case of Wet-w-cont-NV. This is ascribed to the fact that the pre-charged airborne bacteria strike the media first in case of Wet-w/o-cont-NV as the polyimide films on the EPC electrode were fully covered with the media. This is different from the other three cases using the containers, where many of the bacteria can strike the containers first.

**Figure 3:**
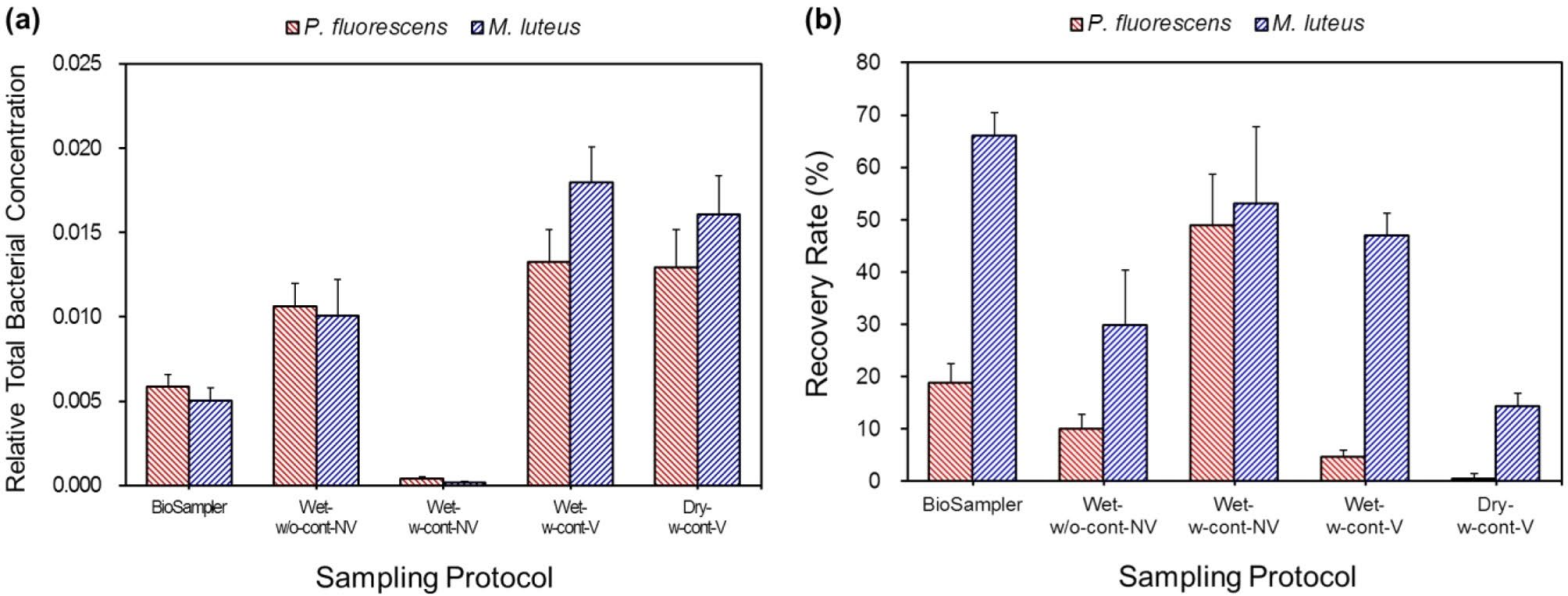
Relative total bacterial concentrations (**a**) and recovery rates (**b**) of *P. fluorescens* and *M. luteus* eluted into sampling media of 1 × phosphate buffered saline in the EPC (− 5 kV) and in the BioSampler. Four different sampling protocols were used in the EPC. *Wet-w-cont-NV* wet sampling with a container, but no vortexing after the sampling, *Wet-w-cont-V* wet sampling with a container and vortexing after the sampling, *Wet-w/o-cont-NV* wet sampling on a polyimide film of the EPC electrode with no vortexing, *Dry-w-cont-V* dry sampling with a container and subsequent vortexing after buffer addition.

Dry-phase electrostatic sampling has been often used for bacterial aerosols^3,6–8,11,12^. In that case, a liquid is added onto the collection spot, and a shear force such as vortexing is applied to extract the deposited bacterial cells. In this study, both the intrinsic collection efficiencies and relative total bacterial concentrations (RTBCs) of the two bacteria, *P. fluorescens* and *M. luteus*, were similar between the cases of Dry-w-cont-V and Wet-w-cont-V (*p* > 0.132), except for the collection efficiencies of *P. fluorescens* (*p* = 0.046) (Figs. 2, 3a). Moreover, many of the airborne bacteria deposited on the containers were re-suspended into the sampling media via vortexing, and hence the amount of intrinsic collection was higher when using a container and subsequent vortexing (Fig. 2). Vortexing was needed to detach the bacteria attached to the containers; however, it also affected the recovery rate of *P. fluorescens*, which can be seen from the comparison between Wet-w-cont-V and Wet-w-cont-NV cases*.*

The recovery rates of the two bacteria in the Dry-w-cont-V case were remarkably lower than those of the Wet-w-cont-V case (*p* < 0.001; Fig. 3b). In fact, a negligibly small amount of culturable bacteria was recovered in the Dry-w-cont-V case, which implies high sensitivity to dryness for these species. Mainelis et al*.*^17^ also showed that very few colonies were formed from electrostatically collected *P. fluorescens* on an agar plate, which was attributed to the significant desiccation stress during continued exposure to air flow. Hydration of bacteria during sampling is thus critical and highly recommended if they are analyzed by culturing methods after sampling. The recovery rates of gram-positive *M. luteus* were higher than those of gram-negative *P. fluorescens* for all the cases, showing better resistance to environmental stresses that can encounter in electrostatic sampling and vortexing.

Another explanation for the higher inactivation of bacteria in the dry-phase sampling (Dry-w-cont-V) compared to the wet-phase sampling (Wet-w-cont-V) may be due to higher adhesion of highly charged bacteria to the dry surface of the containers with opposite polarity. The Hamaker constant between bacteria and a surface is higher without liquids, and hence higher adhesion force can occur between the dry surfaces of bacteria and the container^27^. This would make bacterial membrane deformed to increase the contact areas to the surfaces, and therefore, higher shear stress can be applied to the cells when detaching the deformed cells from the surface using vortexing in the dry-phase sampling compared with the wet-phase sampling. High shear stress and high adhesion force are known to significantly decrease viability of bacteria^28,29^. In fact, specific intracellular reactive oxygen species increased with the shear stress on *Bacillus subtilis*, through possible activation of a plasma membrane bound enzyme such as NADH oxidase, resulting in apoptosis-like programmed cell deaths^28^. Similarly, the recovery rate of *P. fluorescens* was higher in case of Wet-w-cont-NV than Wet-w-cont-V (*p* = 0.003), showing the effects of vortexing on the viability of the bacteria. This implies that the bacteria stuck to the surface were more inactivated compared with the suspended or weakly bound ones. The difference in viability may result from the higher shear stress caused during the detachment of deposited bacteria via vortexing. The suspended or weakly bound bacteria can be easily extracted by gentle pipetting; hence no significant shear stress can be applied to them.

The RTBCs of *P. fluorescens* and *M. luteus* were 2.3 and 3.6 times higher, respectively, in the EPC (Wet-w-cont-V) than the BioSampler despite the lower intrinsic collection efficiencies and sampling flow rate. This result was due to 40 times smaller amount of sampling media used in the EPC, which was made possible because of small sample velocity (~ 0.34 m/s compared to ~ 313 m/s of the BioSampler) in the EPC^25,26^. Acquiring highly concentrated samples is critical for on-site detection of airborne bacteria because the concentration of bacteria in the air is usually very low, and the higher sample concentration the more quickly and reliably sensors can detect on the spot^25,26^.

A substantial amount of bacteria were deposited via electrophoresis, and inactivation of airborne bacteria occurred during dry-phase sampling and bacterial detachment. This observation was based on a sampling media of 1 × PBS, and different sampling media, which can change the interactions between bacteria and surrounding media, were explored for higher physical collection and biological recovery of bacteria. A sampling protocol of Wet-w-cont-V was used in the following experiments.

### Influences of sampling media

**Figure 4:**
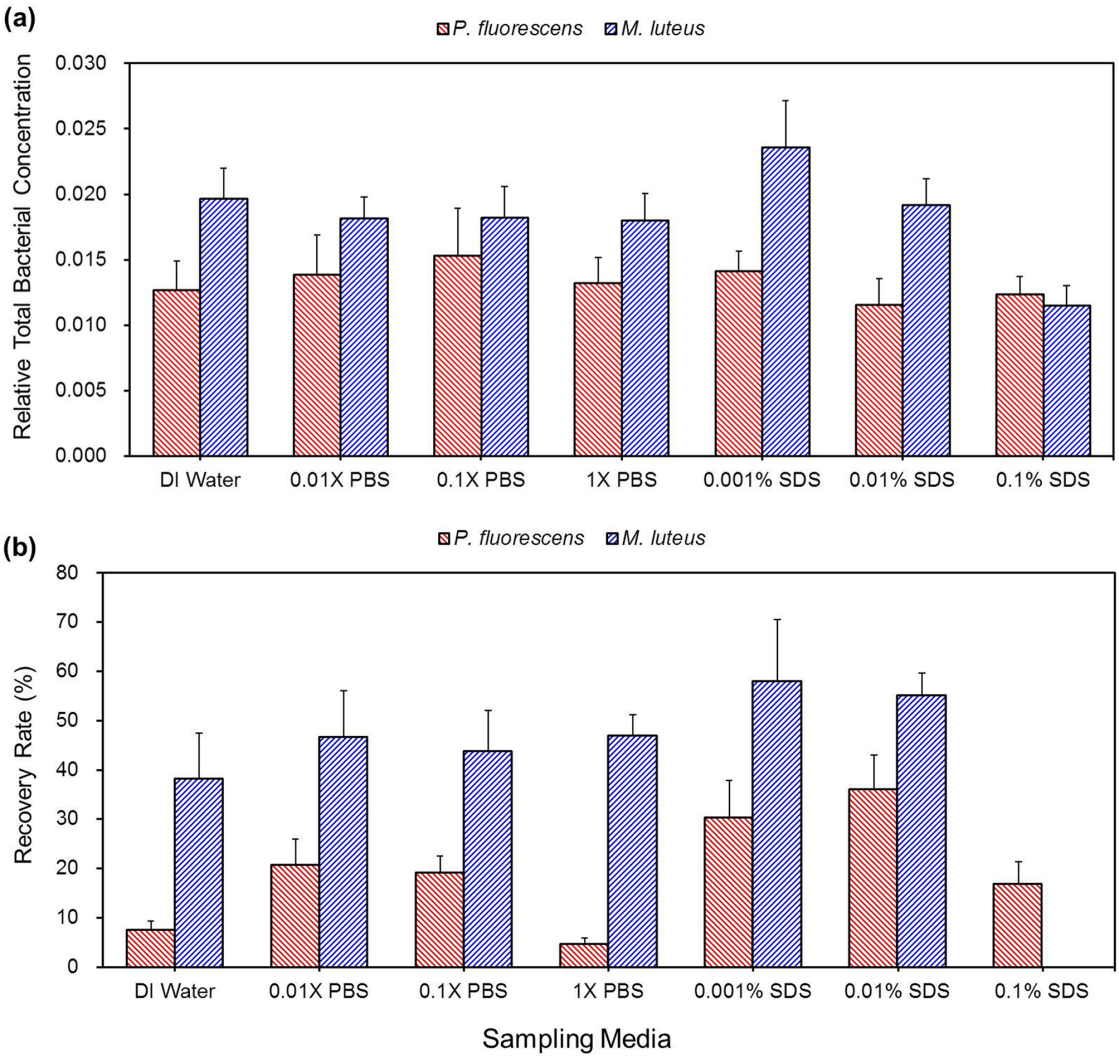
Relative total bacterial concentrations (**a**) and recovery rates (**b**) of *P. fluorescens* and *M. luteus* in the EPC (− 5 kV) for deionized (DI) water, phosphate buffered saline (PBS) and sodium dodecyl sulfate (SDS) diluted with DI water.

Figure 4a shows the RTBCs of the bacteria collected in the EPC (− 5 kV) when PBS, sodium dodecyl sulfate (SDS), and DI water were used as sampling media. The RTBCs of *P. fluorescens* in the EPC were significantly lower than those of *M. luteus* (*p* = 0.01), which can be ascribed to its lower aerosol concentrations relative to those of its initial suspensions (Fig. S1). In fact, the aerosol concentration of *P. fluorescens* through the aerosol pathway was lower than that of *M. luteus* relative to the concentrations of respective initial suspensions (*p* = 0.0262)*.*

The number of bacteria attached to the bottom surfaces of the containers was also quantified immediately before and after vortexing following electrostatic capture (Fig. S2). Almost all *M. luteus* in the containers were attached to the bottom surface whereas some of the *P. fluorescens* in the container were in the media, which may be due to difference in the number of elementary charges attached to the bacteria through corona charging. The measured average number of elementary charges of *M. luteus* and *P. fluorescens* was 547 ± 34 and 427 ± 25, respectively.

It is interesting that the recovery rates of *P. fluorescens* were higher in SDS (except for 0.1%) than in DI water and 1 × PBS (*p* < 0.0002; Fig. 4b), which is commonly used for bioaerosol sampling. The recovery rates of the bacteria from 0.01% SDS were higher than those from the other non-surfactant sampling media tested (*p* < 0.0001; Fig. 4b). This result can be ascribed to reduced adhesion force by the surfactant, considering that cell lyses in this surfactant concentration was negligible during a storage time of up to 30 min (Fig. S3). As the contact areas of bacterial cells are increased with the adhesion force, the shape of the cells as well as the integrities of the membrane can be changed. This stress may result in inactivation of the bacteria^29^. Since gram-negative *P. fluorescens* has a thinner cell wall compared with the gram-positive *M. luteus*, it may be more susceptible to the stress acting on the cell. From these results, intermediate PBS concentration from 0.01 × to 0.1 × and SDS concentration from 0.001% to 0.01% can be suitable for sampling media of airborne bacteria in the EPC. Lower recovery rates of the bacteria in 0.1% SDS were observed, which may be due to lysis of the bacteria during the sampling and storage^30^.

Since sampling media should be appropriate for a long-term storage as well, the time-dependence of the bacterial culturability was investigated in selected sampling media (Fig. S3). The culturable bacterial concentrations did not significantly change after 8-h-storage at 24 °C in DI water, 1 × PBS, and 0.01% SDS, except for *M. luteus* in 0.01% SDS. The culturability of *M. luteus* in 0.01% SDS gradually decreased with the storage time because of the possible damage of the bacterial cells in the detergent solution compared with the other non-detergent ones. Moreover, a significantly lower number of culturable *M. luteus* were recovered from 0.1% SDS compared with those in the other SDS concentrations tested and the other non-surfactant sampling media (Fig. 4b), showing that *M. luteus* is vulnerable to high concentration SDS*.*

### Influences of applied voltage

**Figure 5:**
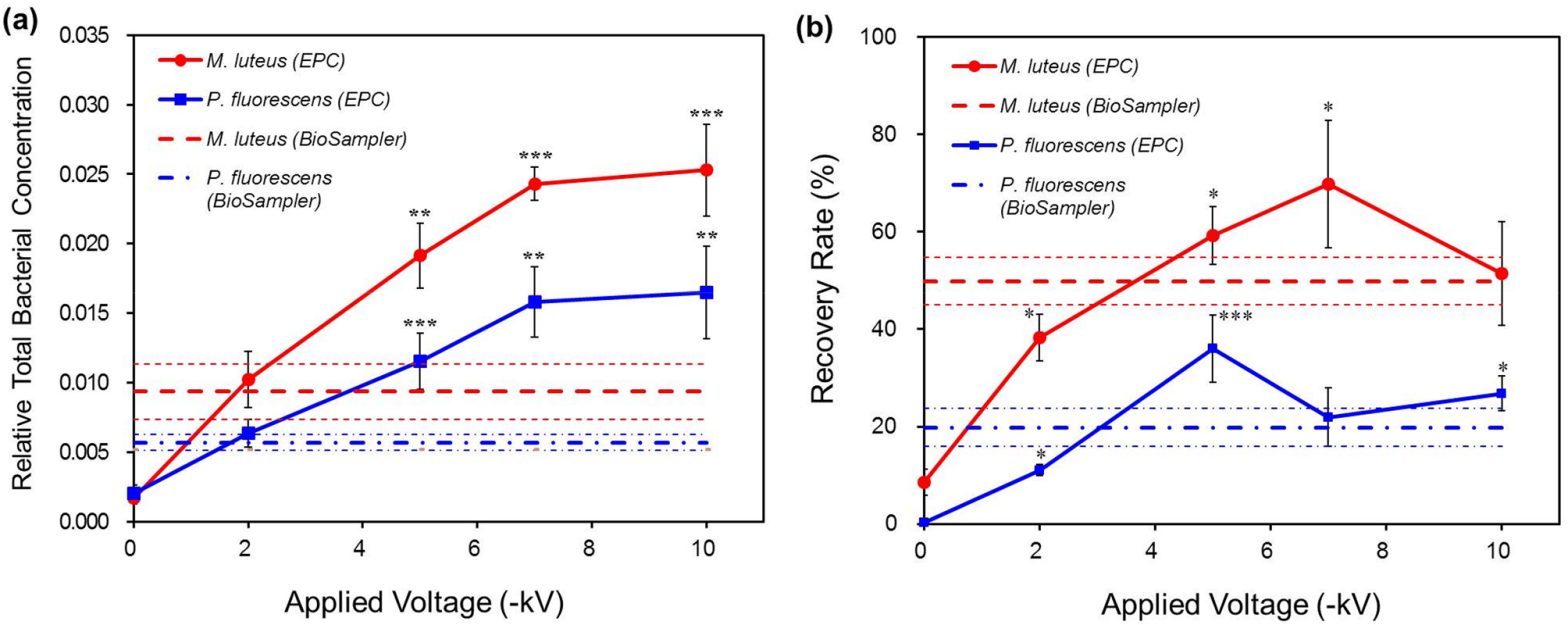
Relative total bacterial concentrations (**a**) and recovery rates (**b**) of *P. fluorescens* and *M. luteus* in the EPC with different applied voltages (from 0 to − 10 kV) and in the BioSampler with a sampling medium of 0.01% sodium dodecyl sulfate. The thinner horizontal lines represent standard deviations of the respective average values in the BioSampler. The statistical differences between the values in the EPC and those in the BioSampler were indicated as asterisks (*p < 0.05, **p < 0.005, ***p < 0.0001).

The effects of applied voltage in the EPC on the recovery of these bacteria were investigated using 0.01% SDS. The RTBCs increased with the increasing magnitude of applied voltage in the EPC because of the increased electric field intensity and attractive force (Fig. 5a). Consequently, the RTBCs of both bacteria were more than 2.7 times higher in the EPC (− 10 kV) than in the BioSampler. Figure 5b shows that the recovery rates of *P. fluorescens* and *M. luteus* in the EPC reached maxima at the applied voltage at − 5 kV and − 7 kV respectively. The lower recovery rates at the lower magnitudes of applied voltage may be due that only highly charged bacterial cells can be collected at the low electric field intensities. Because the viability of airborne bacteria can decrease with increase in their acquired net charges^31^, the recovery rate will be low if many of the collected bacteria are highly charged. On the other hand, the lower recovery rates of the bacteria at higher applied voltages might be due to the cell damage by high electric field intensity^25,32^. Moreover, the adhesion force of the bacteria can also be high at the high electric potential on the surface, thus inducing more stresses to the cells. Abrupt change in their membrane potential might occur at the interfaces between the air and the collection liquid, and between the liquid and the solid surface of the container. Given that the magnitude of applied voltage for the maximum recovery rate was higher for *M. luteus* than for *P. fluorescens*, *M. luteus* is considered more resistant to the stress induced by high electric field. The maximum recovery rates of *P. fluorescens* and *M. luteus* were 36.0% and 69.8% respectively in the EPC, while those in the BioSampler were 19.8% and 49.9% respectively.

## Conclusions

Effects of the sampling media and protocols on the recovery of airborne bacteria were investigated using the personal EPC and the BioSampler towards rapid detection. A substantial amount of bacterial cells were deposited onto the surface of a plastic container or film on the collection electrode of the EPC via the electrophoresis of charged bacteria under electrostatic field. Therefore, we may need to prevent the electrophoretic deposition of particles or to re-suspend the deposited particles into the sampling media. Among the tested sampling protocols, a method of Wet-w-cont-V (Wet sampling with a container and subsequent vortexing after the sampling) offered the most bacteria in the sampling media. Although it is difficult to clarify the exact inactivation mechanisms of the bacteria in the EPC, a few were possible reasons. Adhesion force induced stress can be a cause of the decreased viability of the bacteria, especially for gram-negative *P. fluorescens*, when comparing the cases between (i) wet-phase and dry-phase sampling, (ii) no-vortexed and vortexed bacteria, (iii) surfactants and non-surfactant sampling media, and (iv) intermediate and high applied voltage in the EPC. The recovery rates of *P. fluorescens* and *M. luteus* were significantly higher in the EPC compared with the BioSampler, when 0.01% SDS was used as a sampling medium to decrease the adhesion force of the bacteria. These findings showed the importance of sampling media in electrostatic sampling, and would be valuable in the field of bioaerosol sampling and detection using electrical or electrochemical methods.

## Materials and methods

### Preparation of bacterial suspension

Gram-negative *P. fluorescens* (ATCC 13525) and gram-positive *M. luteus* (ATCC 4698) were used in this study as examples of relatively sensitive and sturdy airborne bacteria respectively^4^. *P. fluorescens* is rod-shaped with the diameter from 0.7 to 0.8 μm and the length ranging from 1.5 to 3.0 μm^33^. *M. luteus* is sphere-shaped and ranges from 0.9 to 1.8 μm in diameter^34^. Tryptic soy broth (TSB) of 20 mL was inoculated with a 100 μL glycerol stock (25%) of *P. fluorescens* and *M. luteus*, and incubated at 160 rpm and 30 °C for 24 and 18 h, respectively. The cultures were centrifuged at 2862*g* (4000 rpm) for 10 min to separate the cells from the media. Resulting pellets were re-suspended in 20 mL DI water and washed 1–2 times by centrifugation at the same condition. The bacterial suspensions for nebulization were made by suspending the washed pellets in 40–50 mL of DI water. The CFU concentrations of *P. fluorescens* and *M. luteus* in the suspensions were 1.85 (± 0.61) × 10^8^ and 2.09 (± 0.80) × 10^8^ CFU/mL, respectively (the values in the parentheses indicate respective standard deviations). The concentrations of *P. fluorescens* and *M. luteus* in the air can be estimated to be 2.45 (± 0.59) × 10^8^ CFU/m^3^ and 2.86 (± 0.63) × 10^8^ CFU/m^3^ respectively, for a sampling medium of 1 × PBS in the EPC (Wet-w-cont-V) by neglecting losses in the recovery rate during aerosolization (Eq. 6).

### Experimental setup

Designs of the EPC and the positive corona charger were published in the previous study^26^, and the experimental setup was constructed for generation, sampling, and measurement of the bacterial aerosols. A polystyrene container (inner diameter: 25 mm, inner height: 4.5 mm, wall thickness: 0.5 mm) was mounted onto the collection electrode of the EPC (Fig. S4), and employed for stable storage of sampling media and vortexing of the collected sample. A smooth and thin film of polyimide tape was attached onto the inner bottom surface of the container for clear fluorescence imaging.

The prepared bacterial suspensions were nebulized by a 3-jet Collison nebulizer (Mesa Laboratories, CO, USA) with 3.0 L/min of clean and dry air. The air was supplied by passing compressed air through a clean air supply (Dekati, Finland) and controlling the flow rate using mass flow controllers (model 5850E, Brooks Instrument, PA, USA). The nebulized bacterial aerosols were dried in a diffusion dryer (HCT, Republic of Korea) and charge-neutralized using a diffusion neutralizer (model 5.622, GRIMM, Germany). The aerosols were then diluted with 10 L/min of the clean and dry air to obtain 13 L/min of bacterial aerosols. The temperature and relative humidity of the bacterial aerosol were measured by a Traceable^®^ hygrometer and they were 24 (± 1) °C and 16% (± 3%) respectively (the values in the parentheses indicate respective standard deviations). The average elementary charges of airborne bacteria were measured with an electrometer (Charme, Palas, Germany). The particle size distributions of the generated bacterial aerosols were measured using an optical particle counter (OPC) (model 1.109, GRIMM, Germany). Measured geometric mean optical diameters of *P. fluorescens* and *M. luteus* were 0.68 and 0.73 μm, respectively.

### Experimental procedure

For the sampling of bacterial aerosols, an air flow rate of 1.2 L/min and a volume of 0.5 mL for sampling media were used in the EPC. A flow rate of 12.5 L/min and sampling media volume of 20 mL were used in the BioSampler as the optimal operating conditions. Applied voltages in the EPC and the corona charger were set to − 5.0 kV and + 3.0 kV, respectively. The sampling time was 10 min for all cases.

Different sampling protocols were used in the EPC in order to investigate the effects of wet sampling and vortexing after the sampling. Here, 0.5 mL of 1 × PBS (0.8% NaCl, 0.02% KCl, 0.061% Na_2_HPO_4_, and 0.019% KH_2_PO_4_) was used as an isotonic sampling medium for bacteria. In case of Wet-w/o-cont-NV, the medium was spread on a thin circular polyimide film (diameter: 24 mm and thickness: 0.2 mm) attached on the collection electrode of the EPC instead of the plastic container. In case of Wet-w-cont-NV, the container was mounted on the electrode, the medium was put in the container, and no vortexing was applied after the sampling. For the case of Wet-w-cont-V, both the container and sampling medium were used, and vortexing was carried out after the sampling. In case of Dry-w-cont-V, dry-phase sampling was conducted, in which the medium was added after sampling for subsequent vortexing. Vortexing in these two cases was carried out for 10 s after covering the container with a polyethylene cap. The collected liquid samples were transferred to micro tubes by gentle pipetting for culture assay and fluorescence microscopy analysis. After sampling, the containers were cleaned for reuse using lint-free wipers with 70% ethanol. The same media were used in the BioSampler for comparison with the EPC.

Wet-w-cont-V was conducted in the EPC for different concentrations of sampling media to investigate their effects on the physical collection and biological recovery of the bacteria. PBS concentrations were varied from 0 × to 1 ×, and SDS (SR1010-100-00, Biosesang, Korea) was used as an anionic surfactant to reduce adhesion and easily detach the deposited particles from the surface of the container. Because high concentration of SDS (~ 1%) is generally used for cell lysis, lower concentrations from 0 to 0.1% were used for the present sampling purpose. PBS and SDS were diluted with DI water to make different concentrations. In order to investigate the suitability of SDS for bacterial storage at 24 °C, time-dependent culturability of the bacteria in 0.01% SDS was measured, and compared with that in DI water and 1 × PBS. Lastly, the effects of applied voltage on the recovery of the bacteria were investigated varying the EPC voltage from 0 to − 10 kV when 0.01% SDS was used as a sampling medium.

### Evaluation of sampling

The “Total Collection Efficiency” was calculated based on the particle number concentration measured by the OPC before the charger and at the outlet of the samplers; however, it does not represent the actual particle collection in the sampling media. The bacterial cells present in the EPC during sampling consisted of the fractions of (i) the cells deposited on the surfaces of the charger and the EPC excluding the collection electrode owing to corona discharge; (ii) the cells deposited on the surfaces of the container or the polyimide film on the collection electrode; and (iii) the cells suspended in the sampling media with or without vortexing in the EPC. The fraction (i) is denoted by the “Charging Loss” in the EPC, and was calculated by subtracting (ii) and (iii) from particle reduction measured by the OPC after the onset of corona charging at an applied voltage of 0 kV. The fractions (ii) and (iii) are denoted by “Loss on the Container” and “Collection in Liquid”, respectively.

The fluorescence microscopic enumeration of the bacterial cells was conducted as follows. The cells in the microtubes and on the surfaces of the containers were stained with 1–2 μM SYTO 9 at 37 °C for 1 h. The fluorescence images were taken using a microscope (Eclipse 80i, Nikon, Japan) and a CCD camera (Cool SNAP HQ2, Photometrics, AZ, USA). For liquid samples, 10 μL of the sample was placed on a 24 × 24 mm^2^ cover glass and covered with another cover glass to make a thin film of microscope specimen, and at least 9 images were taken using a 10 × objective lens. In addition, at least 9 fluorescent images were taken from the surface of the container without peeling off the film using a 20 × objective lens after the collection liquids were removed and the container was air-dried for several minutes. The number of stained cells were counted using an image processing software, image J (National Institutes of Health, MD, USA). The counting was based on the integrated fluorescence intensity of the image divided by that of single cells. The intrinsic collection efficiency was calculated as:

1 $$ {\text{Collection }}\;{\text{in}}\;{\text{liquid}}\;(\% ) = \frac{{{\text{C}}_{{{\text{sample}}}} \times {\text{V}}}}{{{\text{C}}_{{{\text{OPC}}}} \times {\text{Q}} \times {\text{t}}}} \times 100\% , $$

where C_sample_ is the total bacterial concentration in the sampling media measured by the fluorescence microscopy (#/mL), V is the volume of a sampling medium (mL), C_OPC_ is the aerosol concentration measured by the OPC before the charger (#/L), Q is a sampling flow rate (L/min), and t was a sampling period (min). The particle loss on the container was also calculated as following:

2 $$ {\text{Loss }}\;{\text{on }}\;{\text{the}}\;{\text{container}} \;(\% ) = \frac{{{\text{N}}_{{{\text{container}}}} }}{{{\text{C}}_{{{\text{OPC}}}} \times {\text{Q}} \times {\text{t}}}} \times 100\% , $$

where N_container_ is the total number of particles on the surface of the container, measured by the fluorescence microscopy.

The total and viable bacterial concentrations are important performance characteristics of bioaerosol samplers. The RTBC and RCBC (relative culturable bacterial concentration) were calculated by dividing the concentrations in the sampling media by those in the initial nebulizing suspensions:

3 $$ {\text{RTBC}} = \frac{{{\text{C}}_{{{\text{sample}}}} }}{{{\text{C}}_{{{\text{initial}}}} }}, $$

4 $$ {\text{RCBC}} = \frac{{{\text{CFU}}_{{{\text{sample}}}} }}{{{\text{CFU}}_{{{\text{initial}}}} }}, $$

where C_initial_ is the total bacterial concentration in the initial suspension, and CFU_sample_ and CFU_initial_ are the culturable bacterial concentrations in the sampling media and initial suspension, respectively. The CFUs were obtained using the spread plate method with the tryptic soy agar described earlier. Another important factor is the recovery rate, which indicates a degree of preservation of bacterial culturability (or viability) during aerosolization and sampling. The recovery rate was calculated by dividing the RCBC by the RTBC:

5 $$ {\text{Recovery}}\;{\text{rate }}\;(\% ) = \frac{{{\text{RCBC}}}}{{{\text{RTBC}}}} \times 100\% = \left( {\frac{{{\text{CFU}}_{{{\text{sample}}}} }}{{{\text{C}}_{{{\text{sample}}}} }}} \right)\Bigg{/}\left( {\frac{{{\text{CFU}}_{{{\text{initial}}}} }}{{{\text{C}}_{{{\text{initial}}}} }}} \right) \times 100\% . $$

Culturable bacterial concentration in air, CFU_air_ (CFU/m^3^), can be estimated by neglecting losses in the recovery rate during aerosolization, and is given by

6 $$ {\text{CFU}}_{{{\text{air}}}} = \frac{{{\text{CFU}}_{{{\text{sample}}}} }}{{\frac{{{\text{Intrinsic}}\;{\text{collection}}\;{\text{efficiency}} \;(\% )}}{100} \times \frac{{{\text{Recovery }}\;{\text{rate }}\;(\% )}}{100}}} \times \frac{{\text{V}}}{{{\text{Q}} \times {\text{t}} \times 0.001}}. $$

### Statistical analysis

Every experiment in this study was conducted in quadruplicate. The average values of the experimental data are shown in the figures, and their respective standard deviations are indicated as error bars. Statistical difference was examined using the unpaired Student’s *t*-test with Welch’s correction, and a *p*-value (two-tailed) less than 0.05 was considered significantly different.

## Supplementary Information

Supplementary Figures.
