# Supplementary material for: Physical collection and viability of airborne bacteria collected under electrostatic field with different sampling media and protocols towards rapid detection
Source: Sci Rep. 2021 Jul 16;11:14598. doi: 10.1038/s41598-021-94033-7 (PMC8285527; doi:10.1038/s41598-021-94033-7)
Supplement: Supplementary file 1 — Supplementary Figures. [file 41598_2021_94033_MOESM1_ESM.pdf]

Supplementary Information for

Physical collection and viability of airborne bacteria collected under electrostatic field with different sampling media and protocols towards rapid detection

Seongkyeol Hong[1], Myeong-Woo Kim[1], and Jaesung Jang[1,2,*]

[1]Department of Mechanical Engineering, Ulsan National Institute of Science and Technology (UNIST), Ulsan 44919, Republic of Korea

[2]Department of Biomedical Engineering & Department of Urban and Environmental Engineering, UNIST, Ulsan 44919, Republic of Korea

* Corresponding author. *E-mail address*: jjang@unist.ac.kr (J. Jang).

Size distribution of bacterial particles. Figure S1 shows the particle size distributions of the generated bacterial aerosols measured using an optical particle counter (model 1.109, GRIMM, Germany) with 14 size channels, which can measure the particle diameters ranging from 0.25 to 2 µm. Considering that the particles smaller than 0.4 µm in diameter are not intact bacterial cells, but droplets containing the nutrients, salt, and extracellular substances, particles larger than 0.5 µm diameter were used for evaluation of the sampling. The measured geometric mean diameters for *P. fluorescens* and *M. luteus* were 0.68 and 0.73 µm respectively.

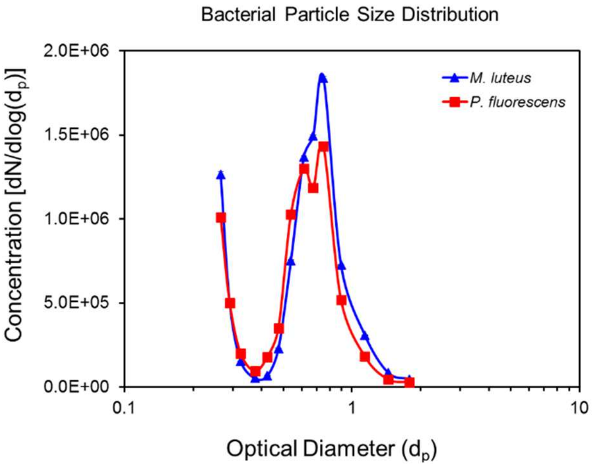

Figure S1. Size distributions of the generated *P. fluorescens* and *M. luteus* particles measured by an optical particle counter (Range: 0.25-2 µm). The particle concentration was normalized by the channel width.

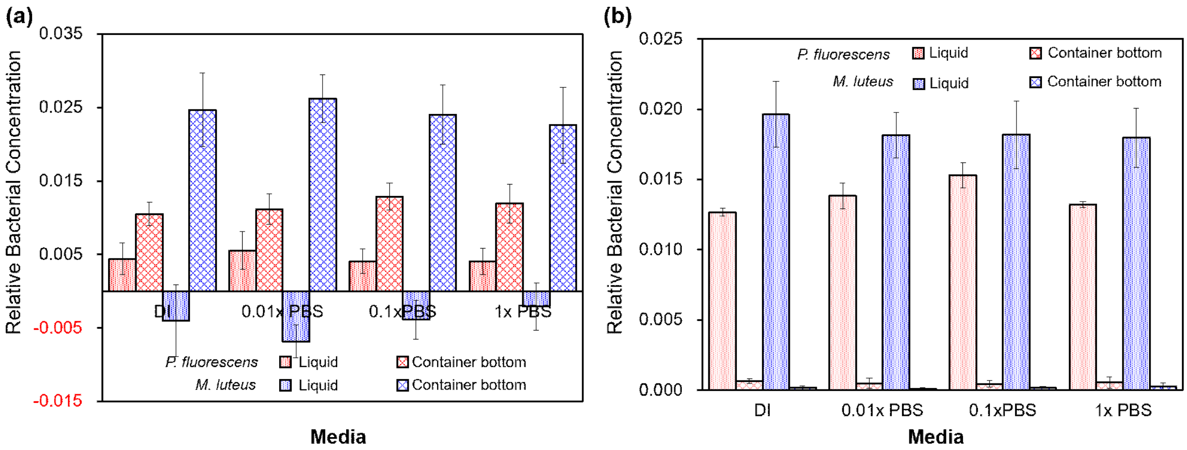

Figure S2. Relative total bacterial concentrations of *P. fluorescens* and *M. luteus* (**a**) before and (**b**) after vortexing in the media and on the bottom of the container of the EPC (-5 kV) for deionized water and different concentrations of phosphate buffered saline (PBS).

Time variation of bacterial culturability in collection liquids. In order to investigate the suitability of sodium dodecyl sulfate (SDS) for bacterial storage, time-dependent culturability of the bacteria in 0.01% SDS was measured and compared with that in deionized (DI) water and PBS. The bacterial suspensions were diluted in 1 mL of the collection liquids in micro-tubes and stored at 24 °C for 8 h. Initial colony forming unit (CFU) concentrations of *P. fluorescens* and *M. luteus* in DI water were 7.68 (± 1.32) × 10^6 and 3.84 (± 0.34) × 10^6 CFU/mL, respectively (the values in the parentheses indicate respective standard deviations), and the CFU concentrations from the collection liquids relative to the initial concentration in DI water were obtained at a storage time of 0–8 h after the collection. The culturable bacterial concentrations were not significantly changed for 8-h-storage in DI water, 1× PBS, and 0.01% SDS, except for *M. luteus* in 0.01% SDS (Fig. S3). The culturability of *M. luteus* in 0.01% SDS was gradually decreased with the storage time because of the possible lysis of the bacterial cells.

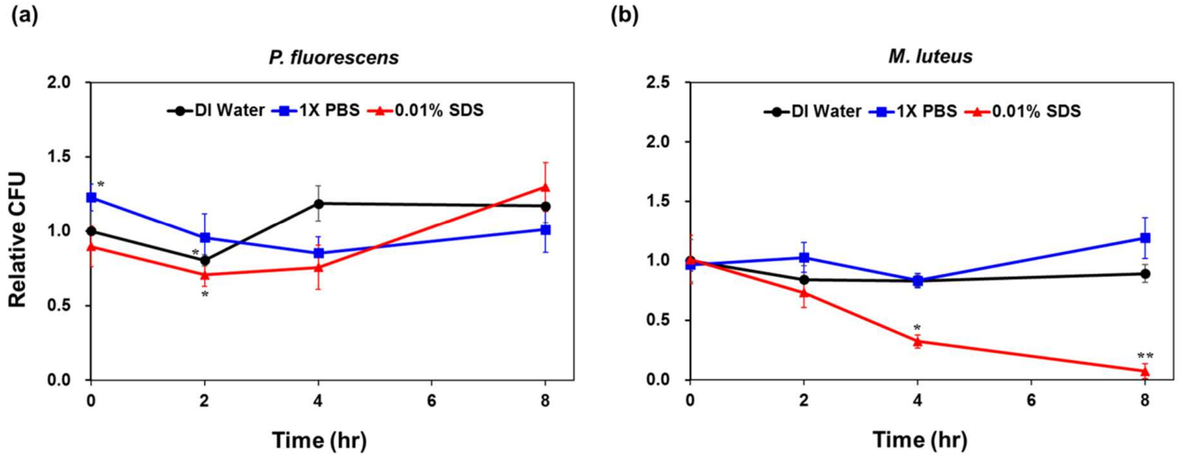

Figure S3. Time-dependent culturability of *P. fluorescens* (**a**) and *M. luteus* (**b**) in three sampling media. The bacteria were stored in deionized (DI) water, 1× phosphate buffered saline (PBS), and 0.01% sodium dodecyl sulfate (SDS) at 24°C for 8 h. Colony forming unit (CFU) concentrations relative to the initial concentration in DI water were shown, and the statistical differences from the reference concentration were indicated by the asterisks (*: $p < 0.05$, **: $p < 0.005$).

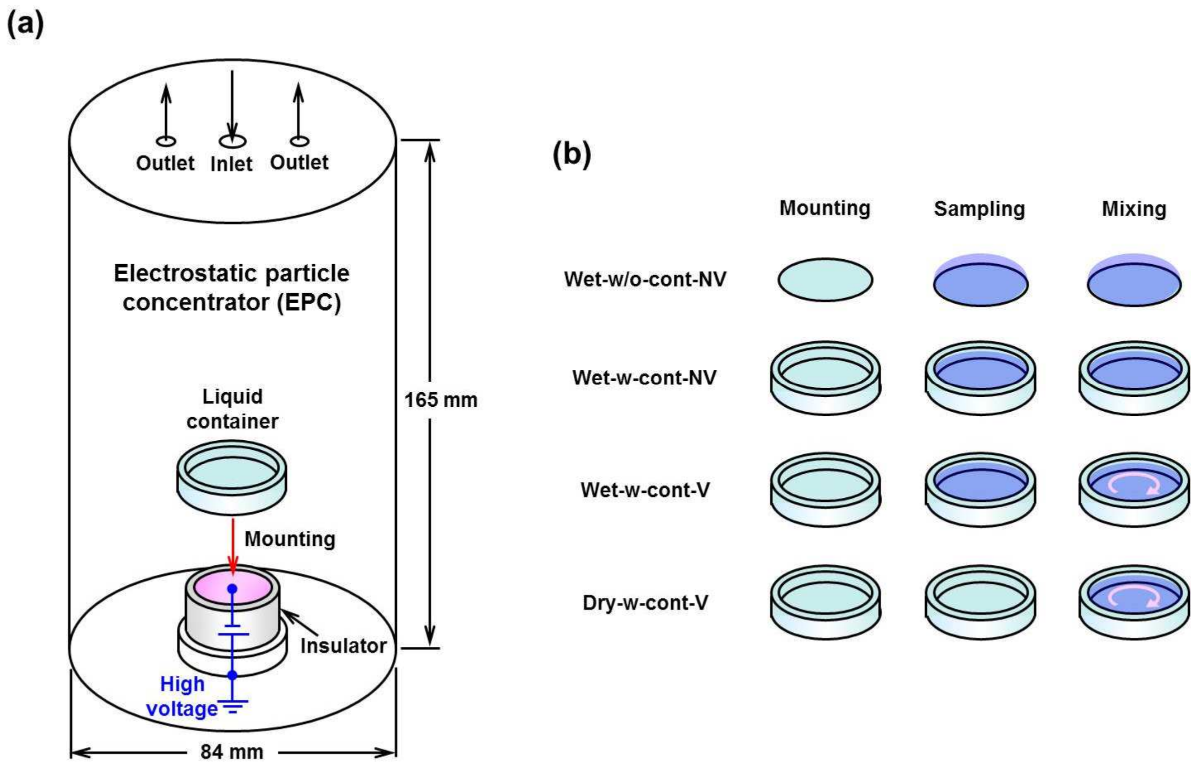

Figure S4. Schematic of the personal electrostatic particle concentrator (**a**) and the sampling protocols (**b**).
